# Supplementary material for: Systematic Analysis of the CCoAOMT Gene Family in Isatis indigotica and the Molecular Mechanism of CCoAOMT8-Mediated Flavonoid Synthesis Under Alkaline Stress Treatment
Source: Biology (Basel). 2025 Oct 30;14(11):1518. doi: 10.3390/biology14111518 (PMC12649902; doi:10.3390/biology14111518)
Supplement: Supplementary file 1 [file biology-14-01518-s001.zip › biology-3907196-supplementary/supplementary Table S2.pdf]

Amino acid sequences of CCoAOMT in 4 species.

>AtCCoAOMT1
MATTTTEATKTSSTNGEDQKQSQNLRHQEVGHKSLLQSDDLYQYILETSVYPREPESMKE
LREVTAKHPWNIMTTSADEGQFLNMLIKLVNAKNTMEIGVYTGYSLLATALALPEDGKIL
AMDVNRENYELGLPIIEKAGVAHKIDFREGPALPVLDEIVADEKNHGTYDFIFVDADKDX
YINYHKRLIDLVKIGGVIGYDNTLWNGSVVAPPDAPMRKYVRYYRDFVLELNKALAADP
RIEICMLPVGDGITICRRIS
>AtCCoAOMT2
MAKDEAKGLLKSEELYKYILETSVYPREPEVLRELRNITHNHPQAGMATAPDAGQLMGM
LLNLVNARKTIEVGVFTGYSLLLTALTLPEDGKVIAIDMNRDSYEIGLPVIKKAGVEHKIDF
KESEALPALDELLNNKVNEGGFXXAFVDADKLNYWNYHERLIRLIKVGGIIVYDNTLWG
GSVAEPDSSTPEWRIEVKKATLELNKKLSADQRVQISQAALGDGITICRRLY
>AtCCoAOMT3
MSTGLALNRCSVSVCRTAVTLLNRPTVSVARSLKFSRRLIGNCSIAPADPYVVADDDKYGN
KQVISLTPRLYDYVLSNVREPKILRQLREETSKMRGSQMQVSPDQAQLLAMLVQMLAAE
RCIEVGVYTGYSSLAVALVLPESGCLVACERDSNSLEVAKRYYELAGVSHKVNVKQGLAA
ESLKSMIQNGEGASYDFAFVDADKRMYQDYFELLLQLVRVGGVIVMDNVLWHGRVSDP
MVNDAKTISIRNFNKKLMDDKRVSISMVPIGDGMTICRKR
>AtCCoAOMT4
MTTFSTSFLFLLLVFCLIGSLAADDLQHKSGRDVCSGGSDLRTPDIRLNRPTDSVVGNCPTE
ASPLVMADDEKYGNKMVISLTPRLYDYVLNNVREHEILKQLREETAISQIQVSPDQAQLLA
MLVEILGAKRCIEVGVYTGYSSLAVALVLPESGRLVACDKDANALEVAKRYYELAGVSHK
VTVKHGLAAESLMSMIQNGEESSYDFAFLDADKAMYQEYFESLLRLVRVGGVIVIDNVL
WHGWVADSTVNDERTISLRNFNKKLMDDQRVSISMVSIGDGMTICRKR
>AtCCoAOMT5
MDGRLPDKGILKSEALKQYIMETTAYPREHELLKELREATIQRYGNLSEMGVPVDESLFLS
MLVKIINAKNTIEIGVFTGYSLFTVALALPEDGRITAIDIDQAGYNLGLEFMKKAGVDHKIN
FIQSDAVRGLDQLLNGKQEYDFAFVDADKTNYVYFLEKLLKLVKVGGIIAFDNTLWFGTL
IQKENEVPGHMRAYREALLEFNKILARDPRVEIAQISIGDGLTLCRRLI
>AtCCoAOMT6
MANEIPTKGILKSEALKQYIMETSAYPREHELLKELRKATVQKYGNLSEMEVPVDEGHFL
SMLVKIMNAKNTIEIGVFTGYSLLTTALALPEDGRITAIDIDKEAYEVGLEFIKKAGVDHKI
NFIHSDGLKALDQLVNDKCEFDFAFADADKSSYVNFHERLLKLVKVGGIIAFDNTLWFGFV
AEDEDGVPEHMREYRAALIEFNKKLALDPRVEVSQISIGDGITLCRRLV
>AtCCoAOMT7
MEKLLPPSKLLPPKGILKSDALKKYIFETTAYPREHEQLKKLREATVLKYGNLSEMEVPVD
EGHFLSMLLKIMNAKKTIELGVFTGYSLLTTALALPHDGHVTGIDIDKEAYEMGLEFIKNA
GVHHKINFIHSDCLQALDNMLSENPKPEFDFAFVDADKPNYANMHERLMKLVKVGGVIA
FDNTLWSGFVAEKEENVPVHMRVNRKAFLDLNKRLAADPHVEVSQVSIGDGVTLCRRLV
>GmCCoAOMT1
MTLIKELEQQPNQIAGHKELAHKSLLQSDALYQYILETSVYPREHESLKELRELTEKHPWN
LMATPPDEGQLLGMLLKLINAKNTMEIGVFTGYSLLSTALALPSDGKAGVAHKIDFREGPA
LPLLDQLIKDEKNKGAFDFIYVDADKDNYLNYHKRVIELVKVGGLVGYDNTLWNGSVVA
PPDAPLMDYVKYYRDFVMELNKALALDSRVEICQLPVGDGITLCRRII

>GmCCoAOMT2
MSSNPVILQSENLTKYILETSVYPREEETLKELRNATASHPWGFMGAAPDAGQLMTLLLK
LLNAKKTIEVGVFTGYSLLLTALTIPDDGKIIALDPDREAYEIGLPFIKKAGVEHKIDFIESPA
LPVLDKLIEDPSNKESFDFAFVDADKDNYWNYHERLLKLVKIGGLIIYDNTLWGGTVAWP
EEDVPAPKRKFRQAALAFNKAIADDSCVEISAVSIGDGFTICRRAH
>GmCCoAOMT3
MDNISKPEVILQSEGLLKYILETGVYPREAEILKELRNATAEHPLGFMGAAPDAGQLMAM
LLKLLNAKKTIEVGVFTGYSLLLTALTIPNDGKIIAMDPDRKAYEIGLPFIKKAGVEHKIDFI
ECPALPVLDKLLEEPANEGSFDFAFIDADKNNYWNYHERLIKLVKIGGLVAYDNTLWGGTV
ALPEKAVSEPKREWRRLSLAFNKAISKDCRVQIAFLSIGDGVIICMRVR
>GmCCoAOMT4
MAEQNQNQTTEAGRHQEVGHKSLLQSDALYQYILETSVYPREPESMKELRELTAKHPWNI
MTTSADEGQFLNMLLKLINAKNTMEIGVYTGYSLLATALALPEDGKILAMDINRENYELG
LPVIKKAGVDHKIEFREGPALPVLDEMIKDEKNHGSYDFIFVDADKDNYLNYHKRLIELV
KVGGVIGYDNTLWNGSVVAPPDAPLRKYVRYYRDFVLELNKALAVDPRIEICMLPVGDGI
TICRRIK
>GmCCoAOMT5
MAEEERHCKSKRGLTKHKMSSNPVILQSVNLTKYILETSVYPREEETLKELRKATAGHPW
GFMGAAPDAGQLMTLLLKLLNAKKTIEVGVFTGYSLLLTALTIPDDGKIIALDPDREAYEI
GLPFIKKAGVEHKIDFIESPALPVLDKLLEDPSNKESFDFAFVDADKDNYWNYHERLLKLV
KIGGLIIYDNTLWGGTVAWPEEDVPVPKRKLRQATLAFNKAIADDSRVEISVVSIGDGFTIC
RRAH
>GmCCoAOMT6
MPKPCCSMHKHYYKTNLANPGPDANRSNQTSHRFEPAFSFLISITLLHPPTSSSSNYQLFQ
KGEEKERKQNAQRIIIAMAEQNQNQTTEAGRHQEVGHKSLLQSDALYQYILETSVYPREP
ESMKELRELTAKHPWNIMTTSADEGQFLNMLLKLINAKNTMEIGVYTGYSLLATALALPE
DGKILAMDINRENYELGLPVIKKAGVDHKIEFREGPALPVLDEMVKDEKNHGSYDFIFVD
ADKDNYLNYHKRLIELVKVGGVIGYDNTLWNGSVVAPPDAPLRKYVRYYRDFVLELNKA
LAVDPRIEICMLPVGDGITICRRIK
>GmCCoAOMT7
MENIKDPSIYRNPVILQSEDLTKYILETAVYPREPAPLKELREATNNHPWGFIATLPEAGQL
MTLLLKLLNPKKTIEVGVFTGYSLLLTALNIPHDGKITAIDINRKTYEVGLPVIKKAGVEHK
IDFIESPALPILDKLLEDPANEGSFDFAFIDADKENYVNYHERLIKLVKIGGLLVYDNTLWG
GRVCWPEDKVPPHARSGRDAAIEFNKTITNDSRVEFALTSVGDGLNICRRVAI
>OsCCoAOMT1
MAATGAGEGKETAAVAGGGGGGGSLHSKTLLKSEPLYQYVLESTVFPREPDCLRELRLATA
NHPMAVMAASPDQVQLFGLLIELISAKNAIEVGVFTGYSLLATALALPDDGKIVAIDVSRES
YDEVGAPVIDKAGVAHKVDFRVGLAMPVLDELVAEEGNKGRFDFAFVDADKVNFLGYH
ERLLQLVRVGGLIAYDNTLWGGSVAAPPAAADEAVPSGRDRSLAALAREFNAAIAADRRV
KPCQLAIADGVMLCRRVA
>OsCCoAOMT2
MPLLVTLLPVYCTAHSRRLKRTTPASRVSSTAMAAANGDASHGANGGIQIQSKEMKTAIHS
NDSPKTLLKSESLHEYMLNTMVYPRENEFMRELRLITSEHTYGFMSSPPEEGQLLSLLLNL
TGAKNTIEVGVFTGCSVLATALAIPDDGKVVAIDVSREYFDLGLPVIKKAGVAHKVDFREG

AAMPILDNLLANEENEGKFDFAFVDADKGNYGEYHERLLRLVRAGGVLAYDNTLWGGS
VALEDDSVLEEFDQDIRRSIVAFNAKIAGDPRVEAVQLPVSDGITLCRRLV
>OsCCoAOMT3
MWGLVDAKLIRVSTAMHRFASASSLPPPAPATAAAAAAQAAALRFGSAATTRVPRALALT
ASTCPWHRRHLCSSSSSSSSAAAAAATAAAVEEARQGRKQLGATTQLYEYLLANVREHPV
LKELREETAAMRGSQMQVSPAQAQLLAMLAQILGAQRCIEVGVYTGYSSLAVALALPESG
RLVACERDERCLEVAKKYYQRSGVAHKVDVKHALAADSLKLLIDGGEVNSYDFAFVDAD
KRMYEEYYELLLKLVRVGGLIVIDNVLWYGRVADPLVNDRKTISIRNFNKKLLEDNRVSIS
MVPIGDGMTICRKLVDT
>OsCCoAOMT4
MAEAASAAAAATTEQANGSSGGEQKTRHSEVGHKSLLKSDDLYQYILETSVYPREHECM
KELREVTANHPWNLMTTSADEGQFLNLLLKLIGAKKTMEIGVYTGYSLLATALAIPDDGT
ILAMDINRENYELGLPSIEKAGVAHKIDFREGPALPVLDQLVEEEGNHGSFDFVFVDADKD
NYLNYHERLMKLVKVGGLVGYDNTLWNGSVVLPADAPMRKYIRYYRDFVLELNKALAA
DHRVEICQLPVGDGITLCRRVK
>OsCCoAOMT5
MATYRPGSNTLLKSDSILEYVLDTTVYPREHERLRELRLITQNHPKSFMGSSPDQMQFFSV
LLKMIGARNAVEVGVFTGYSLLATALALPDDGKVVAIDVSREYYELGRPVIEDAGVAHKV
DFRHGDGLAVLDQLLAGGEGKFDFAYADADKEQYRGYHERLVRLLRVGGVVAYDNTLW
GGSVAMPRDTPGSSAYDRVVRDYMVGFNAMVAADDRVEACLLPVADGVTLCRRLK
>OsCCoAOMT6
MTTGNGDAPVIKNAHSDIDSTNKTLLKSDALYKYVLDTTVLPREPECMRDLRLITDKHQ
WGFMQSSADEAQLLGMLLKMAGAKRTIEVGVFTGYSLLATALALPEDGKVVAIDPDRES
YEIGRPFLEKAGVAHKVDFREGKGLEKLDELLAEEAAAGREAAFDFAFVDADKPNYVKY
HEQLLQLVRVGGHIVYDNTLWAGTVALPPDTPLSDLDRRFSVAIRDLNSRLAADPRIDVCQ
LAIADGITICRRLV
>DfCCoAOMT1
MQSNGGEEQTRHQEVGHKSLLQSDALYQYILETSVYPREPECMKELREITAKHPWNIMTT
SADEGQFLNMLLKLTGAEKTMEIGVYTGYSLLATALAIPQDGTVSSWHPCTRILAMDINR
DNYELGLPCIEKAGVAHKIDFREGPALPVLDHLLEDKNLGSFDFVFVDADKDLNYHERLM
KLVRVGGLVGYDNTLWNGFVVLPAEAPKRKYIRYYRDFVLQLNKALADDDRVEICQPPV
GDGITLCRRVK
>DfCCoAOMT2
MAAADAGEGKKTAAGSSLHSKTLLKSEPLYQYILESTVFPREPDCLRELRLATAKHPMAR
MAASPDQVQLFGLLIEVLGARNAVEVGVFTGYSLLATALALPDDGKIVAIDVSRESYDEI
GSPVIEKAGVAHKIDFRVGLALPVLDQLVAEEGNKGRFDFAFVDADKVNFPNYHERLLQL
VRVGGLIAYDNTLWGGSVAVSADEPLSERDRALAGLAREFNAAIANDRRVQVCQLAISDG
VMLCRRVA
>DfCCoAOMT3
MPRFAVASSSLPPSVAAAAALGFGSRHPRVLQSLTLAAAPPRRGSPPWSRRLCMCSSSSS
STAAAVEEARRGRKQLGMTPQLYGYLLANVREHPILRELRQETAAMRGSQMQVSPAQAQ
LLAMLAQILGAQRCIEVGVYTGYSSLAVALALPESGRLVACERDERCLEVAKKYYQRAGV
AHKVYVKHALAADSLRSLLDCGEASSYDFAFVDADKRMYEEYFELLLKLVRVGGLIVMD
NVLWYGRVADPLVNDPKTISIRNFNKRLLEDKRVNISMVPIGDGMTICRKLVDT

>DfCCoAOMT4
MRAIFPSTWRLPPATMPRFAVASSSLPPAAAAAAALGFGATPPCVLQAQTLAAAAPPPPW
RRRLCMCSSSSTAAAVEEARRGRKQLGMTPQLYDYLLANVREHPILRELREETAAMRGSQ
MQVSPAQAQLLAILAQTLGAQRCIEVGIYTGYSSLAVALALPESGRLVACERDERCLEVA
KKYYQRAGVAHKVINICLFLLDEVDVKHSLAVDSLKLLLDCGEAFSYDFAFVDADKRMY
EEYFELLLKLVRVGGLIVMDNVLWYGRVADPLVPIGDGMTICRKLVDTPLPRSSSQ
>DfCCoAOMT5
MANGGDSVPNVHSDSDNDSNSHTLLKSDALYKYVLDTTVLPREPECMRDLRLLTDKHK
WGFMASSPDEAQLLGMLIKMAGAKNTIEVGVFTGYSLLATALALPDDGKVVAIDYDRES
YEAGRPFIEKAGVAHKVDFREGAGLERLEELLAEEAAAGHEARFDFAFVDADKPNYVRY
HEQLLRLVRVGGTIVYDNTLWGGTVALPPDAPMSDLDRRISVAIRDLNAKLAADPRIEVCQ
LTIADGVTICRRLV
>DfCCoAOMT6
MAANGDAGGEVKDIHTNDSTKTLLKSDALHEYMLNTMVYPREHECLRELRLITKEHTYG
FMSSPPDEALLLSLLLKVMGARNTIEVGVFTGCSVLATALAIPDDGKVVAIDVSREYFDLG
LPVIEKAGVAHKVDFREGPARPILDELLADEGNVGKFDFAFVDADKGNYVNYHEQLLRLV
RVGGVLAYDNTLWGGSVALPDDTPLTEDDREIRGSFRAFNAKIAADQRVEAVQLPVADGI
TLCRRVA
>DfCCoAOMT7
MYILLYPENIFPITKARFVDADKPNYVRNHEQLMRQHAVGRHGGKNVWLPMQNGCKHA
NVWCSMVSSPDEARLLGMLIKMTGAKNTIEIVVFTGHSVLATALALPDDGKVVAIDVSRE
YFDLGLPVIQKAGVAHKVDFREGAALARLDELLAEEGNEARFDFAFVDADKPNHVRYHE
QLLRLVRVGGTIMYDNTLWGGTVVLPPDAPMSDLDRQRSAEIRDLNAKLAADKRVEVC
MLAIADGVTICRRLV
>DfCCoAOMT8
MATGGDSVPNVHSNSDSGNKTLLKSEALFQYILDTMVLPRETECMRELRLLTDKHERCS
MVSSPDEAQLLGMLIKMTGAKNTIEVGVLTGHSLLATALALPDDGMVVAIDVSREYFDLG
LPVIQKAGVAHKVDFREGAGLARLDELLTEEGNEARFDFAFVDADKPNHVRYHEQLLRL
VRVGGGMVVLPPDAPMSDLDRQRSAEIRDLNAKLSPVLFRLIF
>DfCCoAOMT9
MATGGDSVPNVHSDIDSSNKTLLKSDALYKYVLDTTVLPREPECMRDLRLLTDKHKWGF
MQSSPDEAQLLGMLIKMAGAKNTIEVGVFTGYSLLATALALPDDGKVVAIDYDGESYEV
GRPFVKKAGVAHKVDFREGSGLDRLDELLAEEAAAGHEARFDFAFVDADKPNYVRYHE
QLLRLVRVGGTIVYDNTLWGGTVALPPDAPMSDLDRRFSVAIRDLNAKLAADPRIEVCQL
AIADGVTICRRLV
>DfCCoAOMT10
MATGGDSVPNVHSDIDSSNKTLLKSDALYKYVLDTTVLPREPECMRDLRLLTDKHKWGF
MQSSPDEAQLLGMLIKMAGAKNTIEVGVFTGYSLLATALALPDDGKVVAIDYDGESYEV
GRPFVKKAGVAHKVDFREGSGLDRLDELLAEEAAAGHEARFDFAFVDADKPNYVRYHE
QLLRLVRVGGTIVYDNTLWGGTVALPPDAPMSDLDRRFSVAIRDLNAKLAADPRIEVCQL
AIADGVTICRRLV
>DfCCoAOMT11
MSTYRPDSKTLLKTDSIWEYVLDTTVYPREHERMRELRLITKEHPRSIWASSPDQMQFFS
VLLKMIGAKNTIEVGVFTGYSLLATALALPADGKVVAIDVNREYYELGRPVIEKAGVAHK

VDFREGDGLTVLDRMLAEDGAGAVAGTFDFAYADAIKMHYGGYHERLLRLVRVGGVIAY
DNTLWGGSVAMPRDTPSSSENDRVARGYIIEFNAMVAADDRVEACLLPFADGVTLCRRLK
>DfCCoAOMT12
MATHSDSVPNVHSNIDSSNKTLLKSEALYQYILDTTVLPREPECMRELRLLTVKHERCNM
ASSPDEAQLLGMLIKMTGAKNTIEVGVFTGHSLLATALALPDDGKVVAIDVSREYFDLGL
PIIRKAGMAHKVDFREGPGLARLDELLAREASAGHEARFDFAFVDADKNNYVRYHEQLL
RLVRVGGTIVYDNTLWGGTVALPPNVPLSDLDRSRSAEIRDFNAKVAADKRVEVCQLAIA
DGVTICRRLE
>DfCCoAOMT13
MAANSDAAGEVKDIHTNGSTKTLLKNDALYEYMLNTMVYPREHECLRELRNTIEVGVFT
GYSVLATALAIPDDGKIVAIDVSREYFDLGLPVIEKAGVAHKVDCDDPHYYHWVITPVNRL
LFEGNEGKFGFAFVDADKGNYGNYHEQLLRLVRVGGVLAYDNTLWGGTVALPDDTPLTE
DDREIRDFIRAFNAKIAADPRVEAVQLPVADGITLCRRVA
>DfCCoAOMT14
MATATADATTATKEQTSGGGGGGEQKTRHSEVGHKSLLQSDALYQYILETSVYPREHECM
KELREVTAKHPWNLMTTSADEGQFLNMLLKLIGAKKTMEIGVYTGYSLLATALAIPEDGT
ILAMDINRENYELGLPCIEKAGVAHKIDFREGPALPVLDQLLEDEANHGSFDFVFVDADK
DNYLNYHDRLMKLVKVGGLVGYDNTLWNGSVVLPADAPMRKYIRYYRDFVLELNKALA
ADERVEICQLPVGDGITLCRRAK
>DfCCoAOMT15
MATATADATTATKEQTSGGGGGGEQKTRHSEVGHKSLLQSDALYQYILETSVYPREHECM
KELREVTAKHPWNLMTTSADEGQFLNMLLKLIGAKKTMEIGVYTGYSLLATALAIPEDGT
ILAMDINRENYELGLPCIEKAGVAHKIDFREGPALPVLDQLLEDEANHGSFDFVFVDADK
DNYLNYHDRLMKLVKVGGLVGYDNTLWNGSVVLPADAPMRKYIRYYRDFVLELNKALA
ADERVEICQLPVGDGITLCRRAK
>DfCCoAOMT16
MATATADATTATKEQTSGGGGGGEQKTRHSEVGHKSLLQSDALYQYILETSVYPREHECM
KELREVTAKHPWNLMTTSADEGQFLNMLLKLIGAKKTMEIGVYTGYSLLATALAIPEDGT
ILAMDINRENYELGLPCIEKAGVAHKIDFREGPALPVLDQLLEDEANHGSFDFVFVDADK
DNYLNYHDRLMKLVKVGGLVGYDNTLWNGSVVLPADAPMRKYIRYYRDFVLELNKALA
ADERVEICQLPVGDGITLCRRAK
>DfCCoAOMT17
MRAIFPSTWRLPPATMPRFAVASSSLPPAAAAAAALGFGATPPCVLQAQTLAAAAPPPPW
RRRLCMCSSSSTAAAVEEARRGRKQLGMTPQLYDYLLANVREHPILRELREETAAMRGSQ
MQVSPAQAQLLAILAQTLGAQRCIEVGIYTGYSSLAVALALPESGRLVACERDERCLEVA
KKYYQRAGVAHKVDVKHSLAVDSLKLLLDCGEAFSYDFAFVDADKRMYEEYFELLLKL
VRVGGLIVMDNVLWYGRVADPLVNDAKTISIRNFNKKLLEDKRVNISMVPIGDGMTICRK
LVDTPLPRSSSQ
