# Supplementary figures and images for: Systematic Analysis of the CCoAOMT Gene Family in Isatis indigotica and the Molecular Mechanism of CCoAOMT8-Mediated Flavonoid Synthesis Under Alkaline Stress Treatment
Source: Biology (Basel). 2025 Oct 30;14(11):1518. doi: 10.3390/biology14111518 (PMC12649902; doi:10.3390/biology14111518)

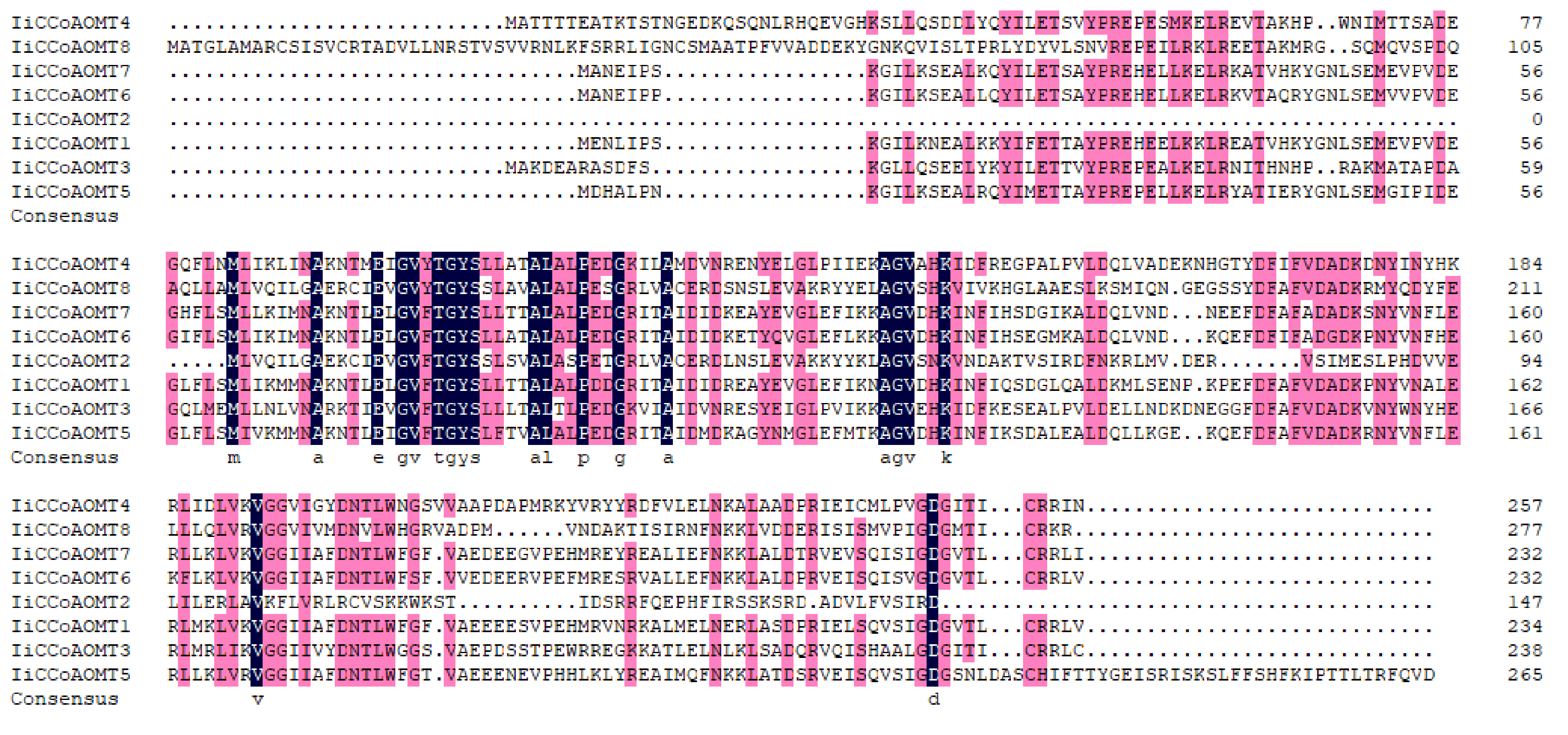

Supplement: Supplementary file 1 [file biology-14-01518-s001.zip › biology-3907196-supplementary/supplementary Figure S2 Multiple sequence alignment results of IiCCoAOMT proteins..png]
